# An Individualized Postoperative Pain Risk Communication Tool for Use in Pediatric Surgery: Co-Design and Usability Evaluation

**DOI:** 10.2196/46785

**Published:** 2023-11-17

**Authors:** Michael D Wood, Nicholas C West, Christina Fokkens, Ying Chen, Kent C Loftsgard, Krystal Cardinal, Simon D Whyte, Elodie Portales-Casamar, Matthias Görges

**Affiliations:** 1 Department of Anesthesiology Pharmacology & Therapeutics The University of British Columbia Vancouver, BC Canada; 2 Research Institute BC Children's Hospital Vancouver, BC Canada; 3 School of Information The University of British Columbia Vancouver, BC Canada; 4 Patient Partner Vancouver, BC Canada; 5 Centre de recherche Centre Hospitalier universitaire Sainte-Justine Montreal, QC Canada

**Keywords:** eHealth, risk communication, decision aid, pain, individualized risk, surgery, anesthesia, postoperative, risk, co-design, focus group, requirement definition, prototyping, usability, prototype, child, pediatric, decision support, iterative

## Abstract

**Background:**

Risk identification and communication tools have the potential to improve health care by supporting clinician-patient or family discussion of treatment risks and benefits and helping patients make more informed decisions; however, they have yet to be tailored to pediatric surgery. User-centered design principles can help to ensure the successful development and uptake of health care tools.

**Objective:**

We aimed to develop and evaluate the usability of an easy-to-use tool to communicate a child’s risk of postoperative pain to improve informed and collaborative preoperative decision-making between clinicians and families.

**Methods:**

With research ethics board approval, we conducted web-based co-design sessions with clinicians and family participants (people with lived surgical experience and parents of children who had recently undergone a surgical or medical procedure) at a tertiary pediatric hospital. Qualitative data from these sessions were analyzed thematically using NVivo (Lumivero) to identify design requirements to inform the iterative redesign of an existing prototype. We then evaluated the usability of our final prototype in one-to-one sessions with a new group of participants, in which we measured mental workload with the National Aeronautics and Space Administration (NASA) Task Load Index (TLX) and user satisfaction with the Post-Study System Usability Questionnaire (PSSUQ).

**Results:**

A total of 12 participants (8 clinicians and 4 family participants) attended 5 co-design sessions. The 5 requirements were identified: (A) present risk severity descriptively and visually; (B) ensure appearance and navigation are user-friendly; (C) frame risk identification and mitigation strategies in positive terms; (D) categorize and describe risks clearly; and (E) emphasize collaboration and effective communication. A total of 12 new participants (7 clinicians and 5 family participants) completed a usability evaluation. Tasks were completed quickly (range 5-17 s) and accurately (range 11/12, 92% to 12/12, 100%), needing only 2 requests for assistance. The median (IQR) NASA TLX performance score of 78 (66-89) indicated that participants felt able to perform the required tasks, and an overall PSSUQ score of 2.1 (IQR 1.5-2.7) suggested acceptable user satisfaction with the tool.

**Conclusions:**

The key design requirements were identified, and that guided the prototype redesign, which was positively evaluated during usability testing. Implementing a personalized risk communication tool into pediatric surgery can enhance the care process and improve informed and collaborative presurgical preparation and decision-making between clinicians and families of pediatric patients.

## Introduction

Surgery poses a substantial risk for postoperative pain, with roughly 1 in 5 children experiencing pain 12 months following surgery [1], which can have detrimental consequences on their long-term well-being and future care-seeking behaviors [2]. The discovery of factors that increase the risk of postoperative pain (eg, anxiety, poor pain coping skills, and pain catastrophizing) [1-6] and the development of prehabilitation plans (eg, improved nutrition and exercise) [7-11] presents an opportunity to improve postoperative outcomes (eg, reduced length of stay [12] and reduced pain [13]). However, these risk factors and potential mitigation are not always communicated clearly and consistently in line with the information needs of the patients and their families [14]. Consequently, researchers are developing a risk identification and communication tools to improve information-sharing with patients and to improve care [15], with some initial success (eg, improved comprehension of procedure-associated risks) [16]. This work has all been focused on adult patients, but there is a clear need to extend this approach to pediatric surgery [14].

Furthermore, the development of health risk communication tools has typically not applied user-centered design principles [16,17]. Participatory design techniques, such as co-design focus groups, can directly incorporate stakeholders throughout development and ensure end user needs are addressed in the design process [18]. Engaging end users has several benefits: access to tacit knowledge and improved knowledge generation [19]; more profound understanding of user needs through examining visual overviews and written communication between users [20-22]; improved design of research materials; and improved quality of service, including a better fit between user needs and the service provided, and increased trust during participation in clinical trials [23,24]. These benefits can all potentially increase the generalizability of results [25]. Hence, incorporating primary end users’ feedback into the development process is imperative to creating easy-to-use and effective risk communication tools and their successful uptake in clinical practice.

Aligned with the BC Children’s Hospital’s (BCCH) continued priority of improving pediatric pain management [3] and patient-centered care [4], our research program aims to (1) contribute to the efforts to minimize postoperative pain and to reduce pain medication requirements, specifically opioids and (2) generalize from the specific use case of pediatric postsurgical pain to develop a range of risk prediction communication tools pertinent to other clinical scenarios. In a previous study, we established the design requirements for a pediatric postoperative pain risk visualization tool, which guided the development of the initial prototype for an easy-to-use tool targeted at clinicians and family users [26]. Our preliminary prototype design was guided by our expected end users’ requirements and included the nonthreatening and multimodal presentation of risk, an estimation of risk factors’ contribution, and mitigation strategies to decrease the patient’s level of risk [26].

The purpose of this study was to further develop and evaluate our preliminary pediatric postoperative pain risk communication prototype before implementing it into clinical practice. Hence, we aimed to (1) acquire additional design requirements from both sets of expected end users (clinicians and family members) through a critique of our preliminary design and a series of co-design activities and (2) evaluate the usability of the redesigned prototype through role-play based on low- and high-risk clinical scenarios and follow-up with standardized usability questionnaires.

## Methods

### Study Design and Approval

We conducted a two-stage study: (1) small group co-design over 2 sessions, followed by (2) individual usability evaluation sessions. Both stages were conducted with BCCH clinicians and family participants, including parents whose children had previously undergone surgery and adults with lived pediatric surgical experience.

### Participants

We recruited BCCH attending physicians and nurse practitioners via departmental email distribution lists, parents via BCCH patient experience email lists and in-person invitations in the anesthetic care unit, and adults with previous childhood surgery via a provincial research network platform (REACH BC). A trained research team member described the study in detail and acquired written informed consent or web-based informed consent using Research Electronic Data Capture (REDCap, Vanderbilt University) [27,28]. In our report, parents and adults with pediatric surgical experience are collectively referred to as “family participant(s)” to protect their privacy. All participants were remunerated CAD $25 (US $19.50) per hour for their expertise and time. The co-design sessions included a mixture of family participants and clinicians. Usability evaluation sessions were individual, and participants in these sessions had not previously participated in co-design.

### Data Collection

#### Overview

A brief prestudy questionnaire, administered via REDCap, collected participants’ demographic information. A total of 2 research team members with expertise in qualitative methods conducted 5 web-based co-design and 12 usability testing sessions between December 2021 and August 2022 using Zoom (Zoom Video Communications); 2 researchers cofacilitated each session (MDW along with CF or YC). The researchers briefly introduced the session, including an overview of the research program, described the co-design or usability evaluation process to illustrate the intended purpose of each activity, and conducted an icebreaker activity to increase participant comfort. Panels of approximately 3-5 family participants or 3-5 clinicians were targeted for each co-design session; usability evaluation sessions had 1 participant each.

All sessions were conducted web-based, so participants were required to have an internet connection, access to an electronic device with a camera, and proficiency in English. Co-design sessions lasted approximately 60 minutes each, were audio-recorded, and digitally transcribed; usability testing sessions lasted approximately 45 minutes each. Participant names were replaced by sequential identifiers, and transcripts were verified by a research team member (CF or YC).

#### Co-Design Iteration 1

**Figure 1:**
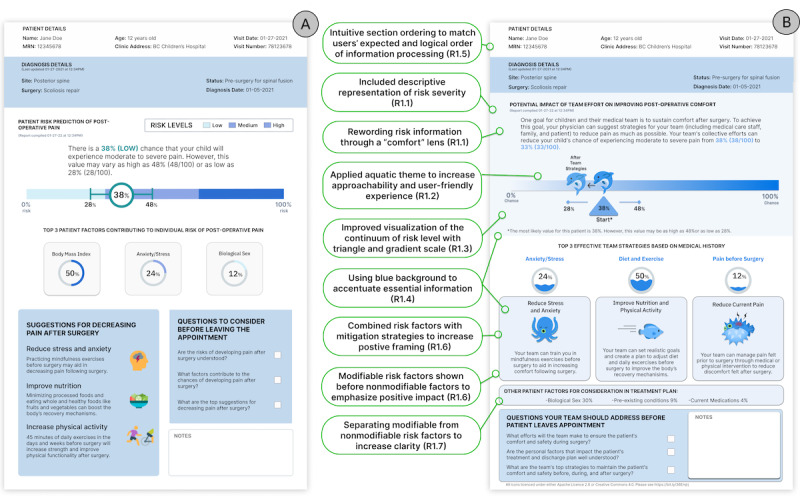
Redesign of the risk score prototype from requirements identified during the first co-design session. (A) The initial prototype [26]. (B) The redesigned prototype. Text in green boxes describes key design requirements identified from thematic analysis (see Table 1).

Participants were given 4 minutes to rapidly sketch 4 distinct pain risk scores to evaluate potential design approaches and visualization strategies. Next, each participant completed a wireframing exercise in turn, in which each participant was presented with empty rectangular text boxes indicating the prototype sections (demographics, risk factors, and mitigation strategies) and risk score examples (bar chart, pie chart, line plot, people count arrays, thermometer, or fuel gauge). They were then instructed to place the 3 prototype sections and 1 risk score into an empty canvas to indicate their preferences for placement, positioning, and sizing of each section. Finally, participants were shown our initial prototype [26] (Figure 1A) and prompted to critically evaluate the design. Data from the first round of co-design sessions were used to generate redesign themes and requirements.

#### Co-Design Iteration 2

Participants were invited to return for a second co-design session to elicit additional design requirements and visualization preferences to inform iterative prototype development. They were also asked to provide feedback on low- and high-risk clinical scenarios (including the corresponding risk score visualization) to be used during the usability evaluation sessions, specifically to comment on the scenario’s accuracy, understandability, realistic representation of patient risk, and whether they had any final suggestions.

#### Usability Evaluation

Pilot usability testing sessions were conducted to confirm the applicability and feasibility of using the proposed low- and high-risk scenarios and to ensure the appropriateness of the interview questions before formal testing sessions. Then, an additional cohort of family participants and clinicians, who did not participate in any previous session, was recruited for usability evaluation.

Each participant was asked to identify information in the various prototype sections, for which we recorded time and accuracy. Specifically, they were asked to complete four tasks: (1) identify key demographic information (patient’s visit number and diagnosis date), (2) report the patient’s likelihood (and uncertainty) of developing postoperative pain if they did not adopt any team strategies to reduce its risk, (3) identify the patient’s risk factor that has the highest contribution to the patient’s overall chance of developing postoperative pain, and (4) indicate what the patient’s chance of developing postoperative pain is if the patient adopts the identified team risk-reduction strategies. This list of tasks was informed by the design team and our patient and clinical partners and was confirmed with study participants during the second co-design session.

Participants were then asked to role-play and “think aloud” through both the low-risk (inguinal hernia) and high-risk (scoliosis correction) scenarios (see Multimedia Appendix 1). Clinicians were asked to state how they would use the tool to communicate risk scores and team risk-reduction strategies to the patients’ families; family participants were asked to state how they would explain their child’s condition to their spouse or child. The order in which the low- and high-risk scenarios were presented to participants was alternated to minimize order effects. Task completion time, the accuracy of prototype interpretation, and the participants’ decision-making processes were recorded and analyzed. At the end of each session, we measured participants’ mental workload with the National Aeronautics and Space Administration (NASA) Task Load Index (TLX) [29] and user satisfaction with the Post-Study System Usability Questionnaire (PSSUQ) [30]. Both were administered via REDCap [28].

### Data Analysis

Session transcripts were analyzed with NVivo, and results were summarized using a thematic analysis [31]. The 2 research team members (MDW along with CF or YC) independently reviewed the 2 transcripts. They used inductive coding [32] to develop a preliminary list of thematic codes organized by theme, subtheme, and participant type [33]. These researchers then compared interpretations and developed consistent codes, which were applied to the remaining 3 transcripts using deductive coding [32]. The 3 researchers discussed additional themes that emerged after coding these remaining transcripts, resolved any discrepancies, and modified the coding framework to ensure that the key concepts were captured. For co-design, a saturation criterion was implemented [34]; specifically, the 2 research team members (MDW along with CF or YC) determined that similar comments and concerns were repeatedly discussed and that data saturation had occurred.

Our prototype was developed using an iterative process in which the research team created, discussed, and revised the prototype using Figma (Figma Inc). Prominent themes emerging from co-design sessions were then used to generate design themes and requirements for prototype redesign.

For the usability evaluation, quantitative data for task completion times, workload, and user perceptions were summarized as median (IQR) values using R (R Core Team). Task accuracies and requests for assistance were summarized as counts and percentages.

### Ethical Considerations

Ethical approval was obtained from the University of British Columbia or Children’s & Women’s Health Centre of British Columbia Research Ethics Board (H20-00613; date of approval 2020-10-20; Principal Investigator: MG). Findings are reported following the Consolidated Criteria for Reporting Qualitative Research checklist [35].

## Results

### Participant Demographics

A total of 12 participants, including 8 clinicians (3 nurse practitioners and 5 anesthesiologists) and 4 family participants attended 5 co-design sessions with a mix of 3-5 clinical and family participants in each session; 12 participants, including 7 clinicians (1 registered nurse, 5 anesthesiologists, and 1 surgeon) and 5 family participants evaluated the usability of our prototype. Family participants were: 6/9 (67%) female; 3/9 (33%) over the age of 49; and 6/9 (67%) with either a certificate (university or nonuniversity) or university degree, and 3/9 (33%) with a high school diploma (or equivalent). Clinicians were female (8/15, 53%), older than 49 years (7/15, 47%), in practice for more than 5 years (10/15, 67%), and clinical fellows (2/15, 13%).

### Co-Design Sessions

#### Overview

Through 2 rounds of co-design sessions, we identified requirements for (1) presentation and usability of the visualization tool, (2) identifying and categorizing risks and mitigation strategies, and (3) supporting collaboration and communication. We present them in detail below.

#### Presentation and Usability of the Visualization Tool

The key themes from the first co-design sessions were that the tool should (A) present risk severity descriptively and visually and (B) ensure that the appearance and navigation are user-friendly (Table 1). Most participants strongly preferred including a descriptive representation of risk severity (from “low” to “high”; design requirement R1.1, Table 1). To represent risk graphically, family participants typically drew child-friendly symbols and real-world objects, such as traffic lights or a race car on a track (R1.2). At the same time, clinicians preferred more traditional graphical representations, such as bar or pie charts (R1.3). This conflict was resolved by incorporating both views in the final set of requirements (R1.2 and R1.3). We were encouraged to change our original circular charts for risk factors and mitigation strategies (Figure 1A) to “fishbowls” for a more child-friendly tone, with the water level representing risk percentage (R1.2; Figure 1B).

**Table 1:** Summary of design requirements obtained from iterative co-design sessions.

Design requirement theme	Requirements elicited during the first co-design session		Requirements elicited during the second co-design session	
	ID	Specific design requirement	ID	Specific design requirement
**(A) Present risk severity descriptively and visually**				
	R1.1	Communicate risk severity descriptively (eg, “low” or “high”)	N/A^a^	N/A
	R1.3	Visualize continuum of risk level		
**(B) Ensure appearance and navigation are user-friendly**				
	R1.2	Use real-world symbols to increase user-friendliness	R2.7	Add page numbers to encourage users to review both pages
	R1.4	Use color to highlight essential information		
	R1.5	Order sections to match users’ intuitive information processing expectations		
**(C) Frame risk identification and mitigation strategies in positive terms**				
	R1.6	Reduce patient and family anxiety by emphasizing positive impact risk mitigation strategies can provide	R2.3	Maintain positive framing of optimizing comfort; include pain terminology in relevant sections
			R2.4	Separate risk scale into before and after to highlight the effect of mitigation strategies
			R2.5	Emphasize mitigation strategies and subsequent effects on reducing the risk of pain
**(D) Clearly categorize and describe risks**				
	R1.7	Increase clarity by improving categorization of risk factors	R2.1	Reword textual risk statements to increase readability and reduce anxiety
**(E) Emphasize collaboration and effective communication**				
	N/A	N/A	R2.2	Emphasize collaboration of cross-functional care teams
			R2.6	Create a separate page for the checklist and notes section

^a^N/A: not applicable.

Participants suggested emphasizing the risk score visualization as it represented essential information. Similarly, the elements for successful risk reduction and the change in risk score “after team strategies” (see also R2.4) were deemed essential and should be emphasized with color (R1.4). The wireframing exercise established an expected ordering of sections (Figure 1B). To accentuate the risk score, most participants enlarged the visualization, placed it centrally in the frame, and placed risk factors and mitigation strategies adjacently (R1.5).

#### Identifying and Categorizing Risks and Mitigation Strategies

Further themes derived from a critical examination of the existing prototype were that the tool should (C) frame risk identification and mitigation strategies positively and (D) clearly categorize and describe risks (Table 1).

Most participants approved of using a risk score’s range with uncertainty, as “it shows that there is always a margin of error” (family participant 2), and showing risk factors with percentages. However, some clinicians insisted that risk information be presented in a way that reduces anxiety, for example, displaying “non-modifiable [risk factors], such as biological sex, might distress the patient” (clinician 7), and “presenting families with a risk assessment of the potential pain after surgery could cause unintended adverse effects…you may be inducing pain by showing patients how much pain they might feel” [Clinician 4].

By “focusing on the possibility of mitigation, clinicians can focus the conversation on discussing pre-habilitation” (clinician 5) to reduce their risk score (R1.6). Separating risk factors that are “modifiable from non-modifiable” (clinician 1) and prioritizing modifiable factors may emphasize positive aspects of improving outcomes (R1.7).

**Figure 2:**
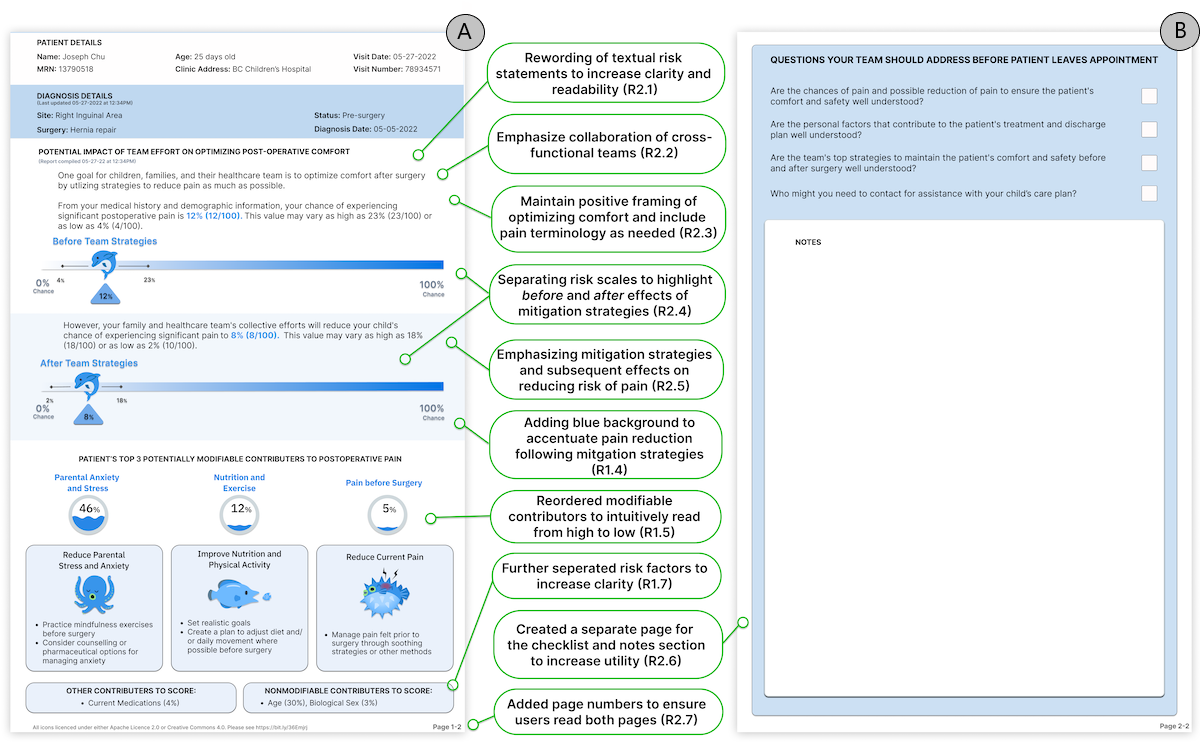
Prototype redesign following the second co-design session. (A) Demographic and clinical information, risk score before and after team strategies, and targeted strategies to reduce risk. (B) Comprehension checklist and expanded notes section on a second page. Text in green boxes describes fundamental redesign changes (see Table 1). For a higher-resolution version of the figure, Multimedia Appendix 2

Following a critical review of the revised prototype in the second co-design session, participants suggested we clarify some textual statements, such as changing “improving comfort” to “optimizing comfort” (R2.1; Figure 2A; for a higher-resolution version, see Multimedia Appendix 2).

Clinicians remained concerned that a patient’s level of pain “is highly influenced by consultation dialogue” (clinician 2) and thus favored using positive framing of risk around the concept of comfort; on the other hand, some family participants insisted that “using comfort/discomfort in a pain management tool confuses the goal of the tool for patients and families” (family participant 4). A clinician noted that “there is a lot of evidence that suggests the language we use can change people’s perception of pain” but acknowledged that some “pain language must be there” (clinician 5). This conflict was resolved by balancing both viewpoints equally in our design requirement; that is, we determined that we should maintain the positive framing of “optimizing comfort” but include pain terminology, such as “significant postoperative pain,” where relevant (R2.3).

Both clinicians and family participants needed clarification about the percentage changes associated with risk reduction (Figure 1B). They suggested we separate our risk score into 2 components: “Before team strategies” and “After team strategies” (R2.4 and see also R1.4). Most participants did not identify the asterisk footnote about the statistical uncertainty of the risk score (Figure 1B), which suggested we move this detail to the textual risk statement (R2.1).

Clinicians and family participants recommended “focusing families on the mitigation strategies” (clinician 2) and their ability to reduce the risk of pain following surgery; that is, we should emphasize how much risk reduction the cross-functional teams can achieve in the “After Team Strategies” risk score (R2.5; Figure 2A). Finally, it was suggested that we reorder the modifiable contributors to read from high to low, reflecting how users would process the information (reinforcing R1.5).

Some participants were confused about whether the risk factor percentages (Figure 1) were population-based or personalized and recommended “stressing that the percentages are individualized” (clinician 4; Figure 2A). Similarly, separating “Other contributors to score” (eg, medication) from “Nonmodifiable contributors to score” (eg, age and biological sex) would improve clarity (reinforcing R1.7).

We were asked to update “Diet” to “Nutrition” to emphasize nutrient intake rather than food quantity (Figure 2A), and a family participant noted that diet and exercise “might be the hardest to implement in your daily life if you have disabilities, live in poverty, and/or a pre-existing condition(s)” (family participant 4). It was suggested that we change the section title to “Potentially Modifiable Contributors” and list the strategies in point form to increase ease of readability (R2.1).

#### Supporting Collaboration and Communication

The final theme from the co-design sessions was that the tool should (E) emphasize collaboration and effective communication (Table 1). Following a critical review of the revised prototype in the second co-design session, participants suggested it should emphasize the collaboration of cross-functional care teams (patient’s family, nurses, and physicians) by changing “your physician” to “your health care team” (R2.2).

Our original notes section was too small; it needed sufficient space for participants to write and draw essential information from the consultation (R2.6) and thus was separated and placed on a separate numbered page (see also R2.7; Figure 2B). Participants approved the checklist questions as reminders of topics to discuss during the consultation (Figure 1B). However, several participants stated the importance of knowing who to contact to assist with their child’s care (see also R2.1). Some insisted that the questions should be connected to the information shown and not require complex answers (see also R2.1; Figure 2B).

### Usability Evaluation

#### Potential Use of the Tool in Practice

During low- and high-risk clinical role-play, participants typically said they would use the tool by comparing the risk scores before and after risk reduction strategies to demonstrate the proposed benefits to motivate positive change. Participants typically said they would describe the top 3 modifiable contributors and ensure the family could implement the targeted strategies. Most participants thought that the comprehension checklist was a helpful reminder and that the notes section was beneficial to document clinical information, such as targeted strategies, referral contacts, aftercare instructions, and questions arising during the consultation.

Most participants agreed that the child would benefit from seeing the tool, at least for older children (>12 years of age), though some felt it would benefit children as young as 5 years of age. There were conflicting views on the use of the tool in practice: some clinicians would not focus on discussing low-risk pain with the patient, or if only a small risk mitigation was anticipated, as it may increase patient anxiety; however, family participants all agreed that the risk score should be used in both low- and high-risk scenarios as any decrease in postoperative pain would be meaningful for a child’s surgical experience. All participants recognized that nonmodifiable contributors, such as biological sex and age, provided context but would not discuss these with the child.

#### Task Completion, Usability Ratings, and Workload

Completion time across tasks varied, but tasks were largely completed quickly (median time to complete each task ranged from median 5.0, IQR 2.9-6.8 seconds to median 16.6, IQR 10.9-34.3 seconds) and accuracy ranged from 11/12 (92%) correct to 12/12 (100%) correct, with only 2 requests for assistance and no evidence for substantial differences between clinicians and family participants (Table 2).

**Table 2:** Participant speed, accuracy, and requests for assistance while identifying essential personalized risk information during usability evaluation.

Task	Time (seconds), median (IQR)			Accuracy (n=12), n (%)		Requests for assistance
	Clinicians (n=7)	Family participants (n=5)				
Task 1: demographics	11.8 (7.5-20.9)	11.4 (9.2-17.4)	11 (92)		0	
Task 2: risk of pain	16.5 (7.2-25.3)	16.6 (15.6-34.3)	11 (92)		1 Family participant	
Task 3: main risk factor	7.0 (3.4-10.7)	6.9 (4.0-9.3)	11 (92)		1 Clinician	
Task 4: effect of team strategies	6.1 (3.0-7.3)	4.3 (2.9-6.1)	12 (100)		0	

Median (IQR) PSSUQ scores [30] (on a 1-7 scale) across all participants were 2.0 (IQR 1.5-2.2) for system usefulness, 2.5 (IQR 1.9-3.0) for information quality, 2.0 (IQR 1.8-2.7) for interface quality, and 2.1 (IQR 1.5-2.7) for overall score, which are within typical ranges of acceptability [36]. The scores were similar between clinicians and family participants, but there was some evidence for differences in their levels of satisfaction with information quality and interface quality (Table 3), which may reflect conflicting views on the use of this tool in all scenarios.

**Table 3:** Post-Study System Usability Questionnaire scores^a^.

PSSUQ^b^ factor	Composite score, median (IQR)			Composite score of ≤3 equating to strongly agree, agree, or somewhat agree, n (%)	
	Clinicians (n=7)	Family participants (n=5)	Clinicians (n=7)		Family participants (n=5)
System usefulness	2.0 (2.0-2.4)	1.3 (1.0-1.5)	7 (100)		5 (100)
Information quality	3.0 (2.7-3.4)	1.7 (1.0-2.0)	4 (57)		5 (100)
Interface quality	2.7 (2.3-3.2)	1.5 (1.0-2.0)	5 (71)		4 (100)^c^
Overall	2.6 (2.2-2.9)	1.4 (1.4-1.5)	5 (71)		5 (100)

^a^Composite scores are the mean of subcomponent scores on a 1-7 scale, with lower numbers indicating greater agreement.

^b^PSSUQ: Post-Study System Usability Questionnaire.

^c^One family participant answered not applicable for all “interface quality” questions.

Median (IQR) NASA TLX subcomponent scores [29] suggested that participants generally felt they were able to perform the tasks required of them, though clinicians scored this subcomponent lower than family participants. Mental demand and effort to achieve their performance contributed the most to the overall workload; scores for temporal demand, physical demand, and frustration were low (Table 4).

**Table 4:** National Aeronautics and Space Administration Task Load Index scores^a^.

TLX^b^ subcomponent	Score, median (IQR)	
	Clinicians (n=7)	Family participants (n=5)
1. Mental demand	27 (19-34)	16 (15-23)
2. Physical demand	12 (0-12)	0 (0-2)
3. Temporal demand	15 (14-18)	3 (1-6)
4. Performance	67 (64-79)	96 (82-97)
5. Effort	50 (17-56)	48 (21-50)
6. Frustration	13 (8-23)	2 (1-3)

^a^Scores are given on a sliding scale from 0=very low to 100=very high.

^b^TLX: Task Load Index.

## Discussion

### Principal Results

In this user-centered study, 5 outline design requirements for a pediatric postoperative pain risk visualization tool were identified during co-design focus group sessions, which built on previously published work [26]: (A) present risk severity descriptively and visually, (B) ensure appearance and navigation are user-friendly, (C) frame risk identification and mitigation strategies in positive terms, (D) categorize and describe risks clearly, and (E) emphasize collaboration and effective communication. A revised risk communication prototype based on these requirements was used by both clinical and family participants quickly and accurately. Only minor frustration was reported during the usability evaluation, and user perception of the tool was acceptable.

### Comparison With Prior Work

#### Development and Use of Risk Communication Tools

Personalized risk communication tools have yet to be widely implemented in clinical practice and have not yet been studied in the context of pediatric pain risk following surgery. However, in preliminary studies previously conducted with adult patients, participants have largely rated personalized risk communication tools as easy to use [37,38], helpful [39], and beneficial to patients [40]. Participants believed that personalized risk communication might result in increased patient engagement [41], increased awareness and understanding of potential surgical complications, and deeper discussion with providers [42]. Participants who were presented with a personalized risk score while consenting for surgery, in particular, agreed that they had received adequate time discussing surgical risks, felt more comfortable with their procedure, had decreased anxiety [43], were significantly more satisfied with the consent process [40,43], and had increased knowledge about the risks associated with their surgery [40]. These other studies explicitly support the motivation behind our research program and the development of a perioperative pediatric risk communication tool. Our usability data mirror some of these findings.

In previous studies, patients also preferred the visual consent tool to text-based documents [42], found personalized surgical risk communication tools helpful for informed decision-making [39], indicated that they would use a personalized risk tool again before a future procedure [40], and believed sharing personalized risk information should be a universal requirement during surgical consultations [44]. Clinicians also highlighted that identifying modifiable risk factors was more important than nonmodifiable ones; thus, separating their contributions was critical [38]. On viewing their risk of postsurgical complications, most patients said they would consider participating in a structured prehabilitation program to decrease their risk and improve postsurgical outcomes [44]. These results are promising and broadly consistent with our findings. However, these previous studies were conducted only with adult participants. While they helped inspire our design decisions, their detailed recommendations can only be fully translated by reevaluating how this approach should be applied in the pediatric surgical setting, as we have done in this study.

#### Personalized and Contextualized Risk Scores

Other studies have shown that data-driven personalized risk calculations can perform significantly better than physicians in predicting patient outcomes in the perioperative domain, and exposure to the calculated risk scores can improve physicians’ prediction accuracy [37]. Patients with low-risk may be more likely to overestimate their surgical risk [39,44], and patients with high-risk may be more likely to underestimate their risk [44], highlighting the importance of improving patient understanding of potential postsurgical complications through supported communication. As we discovered during our usability testing sessions, a point of conflict between patients and health care professionals is that patients typically prefer to be shown their personalized risk scores even when they are low-risk, while some health care professionals felt that this might result in a misuse of clinic time (as was also found in other previous studies [39,41]) or unnecessarily increase anxiety; our family participants universally agreed the risk tool should be used in low- as well as high-risk situations, explaining that any opportunity to decrease postoperative pain, no matter how small, would be beneficial to their child. This further highlights the benefit of involving family participants in the design process to ensure effective use by expected end users.

#### Essential Design and Feature Considerations

Where patients and health care professionals have provided input to the design and feature considerations of personalized risk score tools, their requirements echo the findings from this study. For example, other studies also identified simplicity and clarity as essential characteristics to facilitate shared decision-making [45,46], and to ensure that risk information does not overwhelm patients [45-47]. Other focus group studies have also suggested including less complex language for risk severity, such as “low,” “medium,” and “high” [44], and that simplistic visualizations and language are crucial to understanding—patients have been confused by highly interactive visualizations and better comprehended static charts [45]. These previous findings are similar to the design requirements we established for this prototype.

Patients and care providers have previously indicated that a multimodal risk score supports the learning styles and preferences of various users [40,46]. Furthermore, continuing to engage stakeholders, educating staff, and allowing smooth integration into workflows have previously been noted as important implementation success factors [40]. The findings from these other studies are broadly reflected in our participants’ comments during our co-design sessions and usability evaluation.

#### Differences Between Clinical and Family Participants’ Design Requirements

While addressing user needs overall is critical, we must acknowledge some differences between clinicians’ and family participants’ perceptions of the risk communication tool in our usability testing. While clinicians were satisfied with the tool overall, their lower levels of satisfaction with information and interface quality (as evidenced by their lower PSSUQ scores for these components) may have reflected concerns about what information is presented; clinicians also felt less able to perform the tasks required of them (as evidenced by their lower NASA TLX performance scores). These differences may reflect clinicians’ concerns about the potential adverse effects of communicating risk to patients via the “nocebo effect” [48], which should be recognized and addressed, particularly in the pediatric setting. We incorporated this clinical guidance into the final iteration of our prototype to provide the risk information through a “comfort” lens; that is, we aimed to focus the efforts of the clinician and family team on “maintaining comfort” rather than “reducing pain,” while still acknowledging the risk of pain inherent in their surgical procedure. This approach is consistent with international initiatives aimed at prioritizing compassionate approaches to the recognition, prevention, and treatment of children’s pain (eg, ChildKind International [49]) as well as our own institution’s recommended approach [50]. It seems these changes may not have been sufficient to address the concerns of our clinical participants in usability testing. Importantly, however, other research has shown that, while increased risk has been correlated with patients wanting to discuss the procedure in more detail with their providers [37], presenting patients with their personalized risk has not been associated with canceling procedures or changes in the decision to undergo surgery [39,43,44].

### Limitations and Future Work

We must acknowledge several limitations in our study. First, our study participants included no surgeons during the requirements gathering phase and only 1 surgeon in the usability testing phase; however, all clinician participants were anesthesiologists or nurses with expertise and regular involvement in predicting, diagnosing, treating, and offering families guidance on managing postoperative pain. Second, we did not include children or adolescents. Future work may involve evaluating the benefit of developing a child-centered version of the tool. At this stage, we adopted the strategy of developing a family-centered tool, but we aim to expand this idea to a tool targeted at adolescents. We have begun this process with a perioperative survey of our adolescent surgical population, which evaluates potential preoperative risk factors and postoperative recovery indicators. If successful, we can expand this work to younger children. Participants in this study echoed this aim: most agreed that children older than 12 years of age would benefit, and some felt we could consider including children between the ages of 5 and 11 years.

Third, our focus group sizes were relatively small (3-5 participants per session), typically due to the challenges of scheduling time with clinicians and family participants. While this could have avoided “group think,” it may have hindered collaborative idea generation. Furthermore, we did not use a dynamic prototyping tool in our co-design sessions with participants working collaboratively on a shared resource, which may have enhanced the design process; in pilot-testing, this had been found to be a barrier to participation. However, our patient partners suggested that screen sharing the exercises would increase accessibility as it may be challenging to teach participants of various education and language proficiencies to use a prototyping tool in real time or if they were using a small screen (eg, cell phone) and could not easily complete each exercise, or had a disability that might limit motor function and participation. Fourth, conducting co-design sessions over Zoom rather than in-person may have biased our sample. On the one hand, the requirement for an internet connection may have prevented participation by some families, which may have impacted the social equity of our findings and on the other hand, it may have facilitated participation for some people who would otherwise have been able to contribute due to travel or time constraints.

Finally, our usability evaluation provides only limited evidence of the tool’s readiness for implementation. Although its intended real-world use is as a shared clinician-family resource, we evaluated with individuals (either a clinician or family participant but not both at the same time), using a static prototype, with tasks that may have been too simple to evaluate the usability effectively. That said, the tool is not designed to be a complex, dynamic tool, but rather to communicate a fixed set of information personalized to each clinician-family presurgical encounter.

Future work will involve implementing the design in digital form, which will first be evaluated with a range of clinical scenarios and take account of the need for joint clinician-family evaluation. We are also collecting data in a separate study to generate a risk prediction model, which will supply the personalized risk predictions and proposed prehabilitation strategies that the tool is designed to present [51]. A key point that should be addressed ideally before implementation is how widely the tool should be used in practice: in all cases, as our family participants suggested; only in cases with a high risk of pain; or where team mitigation strategies are expected to make a significant difference.

### Conclusions

This user-centered co-design study identified essential requirements for a pediatric postoperative pain risk visualization tool to present risk severity descriptively and visually, to ensure that appearance and navigation are user-friendly, to frame risk identification and mitigation strategies in positive terms, to categorize and describe risks clearly, and to emphasize collaboration and effective communication. The usability of the resulting paper prototype was positively evaluated by both clinical and family participants suggesting that it is ready to be implemented as a digital prototype that can be tested in a clinical setting to establish its efficacy in supporting communication about postoperative pain risk.

## Appendix

Multimedia Appendix 1 Low- and high-risk scenarios for role-play and "think aloud."

Multimedia Appendix 2 Higher-resolution version of the risk communication tool prototype following the second co-design session.

## Abbreviations

BCCH: BC Children’s Hospital
NASA: National Aeronautics and Space Administration
PSSUQ: Post-Study System Usability Questionnaire
REDCap: Research Electronic Data Capture
TLX: Task Load Index
