# Supplementary material for: An Individualized Postoperative Pain Risk Communication Tool for Use in Pediatric Surgery: Co-Design and Usability Evaluation
Source: JMIR Pediatr Parent. 2023 Nov 17;6:e46785. doi: 10.2196/46785 (PMC10692877; doi:10.2196/46785)
Supplement: Multimedia Appendix 1 [file pediatrics_v6i1e46785_app1.pdf]

An individualized postoperative pain risk communication tool for use in pediatric surgery

Supplementary Appendix 1: low- & high-risk scenarios for role-play and 'think aloud'

Clinician version

Low risk case: pediatric inguinal hernia

Joseph, a 25-day old male came to the pediatric clinic for a swelling in his right groin. Joseph was born at term, and his mother had an uneventful pregnancy and delivery. Joseph is gaining weight and meeting his age-appropriate milestones. His mother noticed the bulge in Joseph's right groin area one week ago that would come and go depending on his position. The pediatrician diagnosed it as an easily reducible right inguinal hernia. The remaining physical examination was unremarkable. A referral was made to a pediatric surgeon who advised repair of the hernia at some point in the next two months depending on operating room availability. The physician enters the necessary details and demographics into our application, which returns the following form…

High-risk case: adolescent idiopathic scoliosis and back pain

Olivia, a 15-year-old, otherwise healthy active female patient presents with lower thoracic back pain. The pain has been increasing in intensity over the past 12 months. Her pain worsens with physical activity, particularly after basketball and soccer games and is not relieved by short courses of acetaminophen (Tylenol), ibuprofen (Advil) and physiotherapy. Her mother has noticed the curve in her spine has increased over the past year. After meeting with their pediatric orthopedic surgeon, the decision was made to proceed with corrective surgery in 6-12 months depending on operating room availability. Olivia reports that she is quite anxious about undergoing surgery, as well as not being able to participate in sports during her recovery. As part of their consultation, the physician enters the necessary details and demographics into our application, which returns the following form…

Parent version

Low risk case: pediatric inguinal hernia

You took your 25-day old son, Joseph, to a clinic for a swelling in his right groin. Joseph was born 40 weeks, and there were no problems with pregnancy or delivery. Joseph is gaining weight and meeting his age-appropriate milestones. You noticed the bulge in Joseph's right groin area one week ago that would come and go depending on his position. Your doctor diagnosed it as an easily reducible right inguinal hernia. The doctor did not find any other problems with Joseph's health in the rest of his physical examination. A referral was made to a pediatric surgeon, who advised the repair of the hernia at some point in the next two months depending on operating room availability. The doctor entered the necessary details and demographics into our application, which returns the following form…

High-risk case: adolescent idiopathic scoliosis and back pain

Your 15-year-old daughter, Olivia, is healthy and active, but has told you that she has pain in her lower back. The pain has been increasing in intensity over the past 12 months. The pain worsens with physical activity, particularly after basketball and soccer games and is relieved by taking Tylenol or Advil or attending physiotherapy. You have noticed a curve in her spine has increased over the past year. After meeting with a pediatric orthopedic surgeon, you have decided to proceed with corrective surgery in 6-12 months depending on operating room availability. Olivia reports that she is quite anxious about undergoing surgery both experiencing significant pain postoperatively and also waking up during surgery, as well as not being able to participate in sports during her recovery. As part of their consultation, the physician enters the necessary details and demographics into our application, which returns the following form…
