# Supplementary material for: An Individualized Postoperative Pain Risk Communication Tool for Use in Pediatric Surgery: Co-Design and Usability Evaluation
Source: JMIR Pediatr Parent. 2023 Nov 17;6:e46785. doi: 10.2196/46785 (PMC10692877; doi:10.2196/46785)
Supplement: Multimedia Appendix 2 [file pediatrics_v6i1e46785_app2.pdf]

PATIENT DETAILS

Name: Joseph Chu
MRN: 13790518
Age: 25 days old
Clinic Address: BC Children’s Hospital
Visit Date: 05-27-2022
Visit Number: 78934571

DIAGNOSIS DETAILS
(Last updated 05-27-2022 at 12:34PM)

Site: Right Inguinal Area
Surgery: Hernia repair
Status: Pre-surgery
Diagnosis Date: 05-05-2022

POTENTIAL IMPACT OF TEAM EFFORT ON OPTIMIZING POST-OPERATIVE COMFORT
(Report compiled 05-27-22 at 12:34PM)

One goal for children, families, and their healthcare team is to optimize comfort after surgery by utlizing strategies to reduce pain as much as possible.

From your medical history and demographic information, your chance of experiencing significant postoperative pain is **12% (12/100).** This value may vary as high as 23% (23/100) or as low as 4% (4/100).

Before Team Strategies

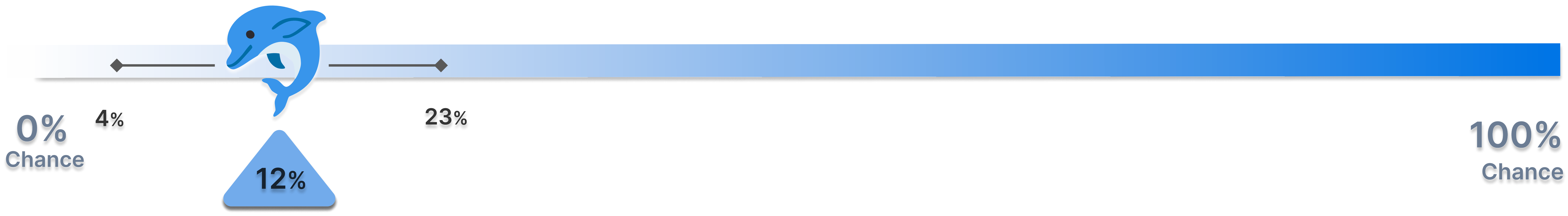

However, your family and healthcare team's collective efforts will reduce your child's chance of experiencing significant pain to **8% (8/100).** This value may vary as high as 18% (18/100) or as low as 2% (10/100).

After Team Strategies

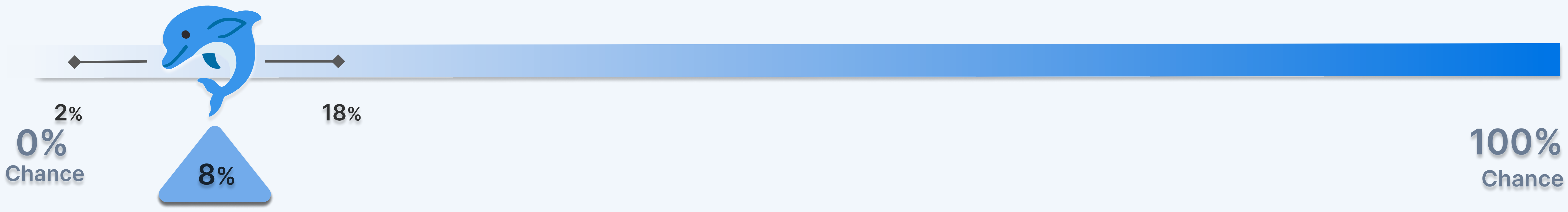

PATIENT’S TOP 3 POTENTIALLY MODIFIABLE CONTRIBUTERS TO POSTOPERATIVE PAIN

Parental Anxiety and Stress

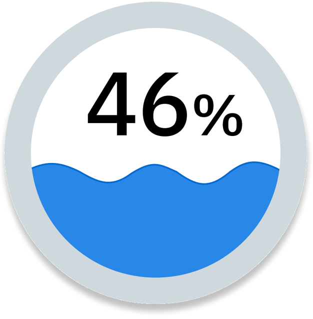

Nutrition and Exercise

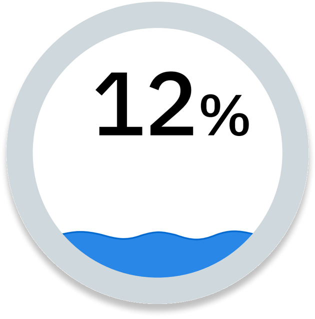

Pain before Surgery

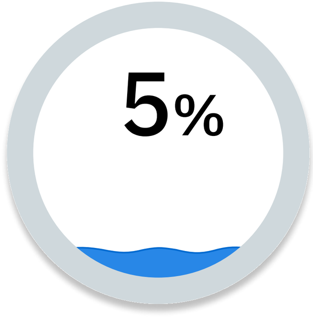

Reduce Parental Stress and Anxiety

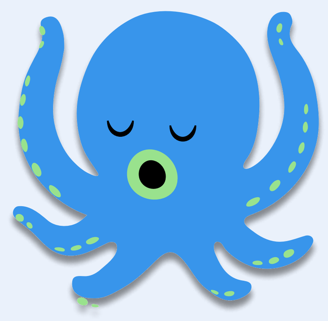

- Practice mindfulness exercises before surgery
- Consider counselling or pharmaceutical options for managing anxiety

Improve Nutrition and Physical Activity

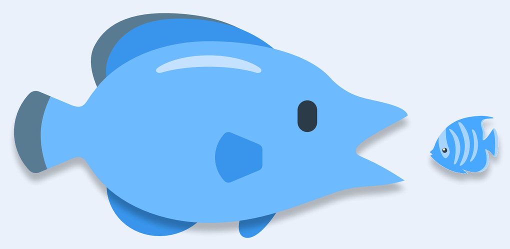

- Set realistic goals
- Create a plan to adjust diet and/ or daily movement where possible before surgery

Reduce Current Pain

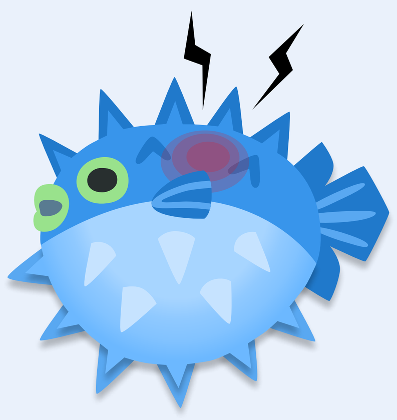

- Manage pain felt prior to surgery through soothing strategies or other methods

OTHER CONTRIBUTERS TO SCORE:

- Current Medications (4%)

NONMODIFIABLE CONTRIBUTERS TO SCORE:

- Age (30%), Biological Sex (3%)

All icons licenced under either Apache Licence 2.0 or Creative Commons 4.0. Please see https://bit.ly/36Emjrj

QUESTIONS YOUR TEAM SHOULD ADDRESS BEFORE PATIENT LEAVES APPOINTMENT

- [ ] Are the chances of pain and possible reduction of pain to ensure the patient's comfort and safety well understood?
- [ ] Are the personal factors that contribute to the patient's treatment and discharge plan well understood?
- [ ] Are the team's top strategies to maintain the patient's comfort and safety before and after surgery well understood?
- [ ] Who might you need to contact for assistance with your child’s care plan?

NOTES
